# Idiopathic Oesophageal Dysmotility Disorder: Stridor Secondary to Megaesophagus

**DOI:** 10.1155/2013/368504

**Published:** 2013-11-27

**Authors:** B. G. Natesh, N. Caton, D. Kim, A. Shetty

**Affiliations:** ^1^Queen Alexandra Hospital, Southwick Hill Road, Portsmouth, Hampshire PO6 3LY, UK; ^2^ENT Department, St. Mary's Hospital, Praed Street Paddington, London W2 1NY, UK

## Abstract

We present an interesting case of an elderly lady who presented with stridor caused by megaesophagus secondary to an acquired idiopathic dysmotility disorder. We discuss the aetiology and management of megaesophagus secondary to this condition and how it differs from megaesophagus secondary to achalasia.

## 1. Introduction

Megaesophagus is commonly associated in patients with achalasia cardia. These patients can present with stridor and this is widely published in the literature. We present an interesting case of an elderly lady who was presented with stridor secondary to an acquired idiopathic dysmotility disorder. We discuss the aetiology and management of megaesophagus secondary to this condition.

## 2. Case Report

A 94-year-old female was presented with a 24-hour history of rapidly increasing stridor associated with a diffuse anterior neck swelling and chest pain radiating to her back. She had been experiencing hoarseness of voice for the past four months. She did not report any history of dysphagia or odynophagia. Her past medical history included a benign tumour of the soft palate, which was treated with radiotherapy during the late 1980's. She is a nonsmoker.

**Figure 1:**
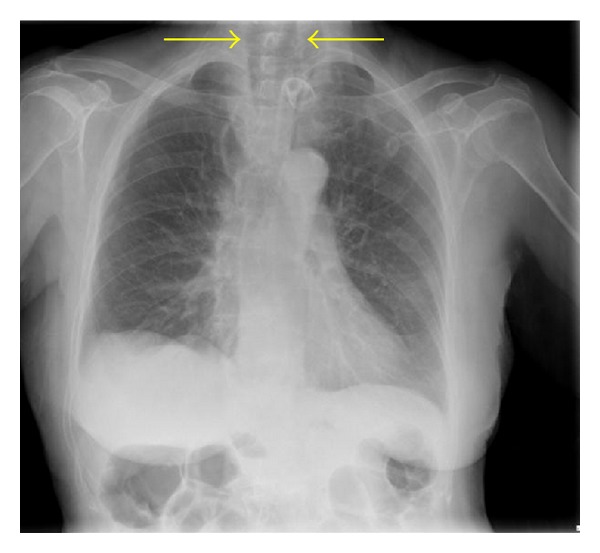
Chest radiograph showing large air shadow in the neck.

On examination in the emergency department, she had a diffuse, firm anterior neck swelling that was nontender. There was obvious distension of the neck and upper anterior chest veins suggesting the possibility of a superior vena cava obstruction as a result of the neck swelling. The patient was in extreme distress with an impending airway obstruction. A chest radiograph (Figure 1) was performed in the emergency department and demonstrated an unusual large air- filled swelling in the neck. It was evident that the patient was tiring and struggling to maintain adequate oxygenation on maximal oxygen therapy. The patient was therefore directly transferred to theatre for further assessment and definitive airway management. In theatre, with attendance of the anaesthetic and ENT teams, a flexible nasoendoscopic examination demonstrated a smooth and diffuse, noninflamed swelling of the posterior pharyngeal wall which was causing an acute airway obstruction. Vocal cord movements were normal but there was severe oedema of the supraglottic tissues, which further compromised the airway.

A secure airway needed to be established so the patient underwent awake fibre-optic intubation, which proved to be difficult given the gross oedema of the upper airway. Given the difficulty with intubation, a decision was agreed to perform an emergency tracheostomy under local anaesthesia. Subsequent rigid pharyngoscopy demonstrated a compressible mass in the posterior pharyngeal wall, and oesophagoscopy revealed massive food impaction of the whole length of the oesophagus within a grossly dilated oesophagus. The impacted food bolus had displaced the upper oesophagus by causing it to “slide” behind the pharynx to create the observed posterior pharyngeal wall swelling. Further, the food impaction had caused local venous congestion and secondary oedema of the upper airway mucosa compounding the airway obstruction. Food was evacuated up to 33 cms, which fully resolved the posterior pharyngeal wall swelling with correction of the airway compromise.

**Figure 2:**
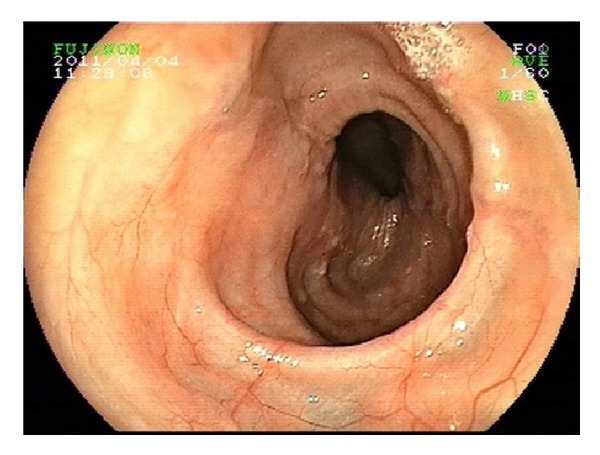
Gastroscopy showing dilated and tortuous oesophagus.

**Figure 3:**
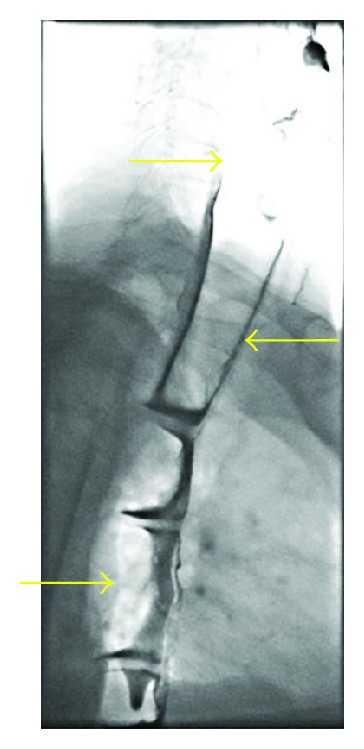
Barium swallow showing dilated midoesophagus.

**Figure 4:**
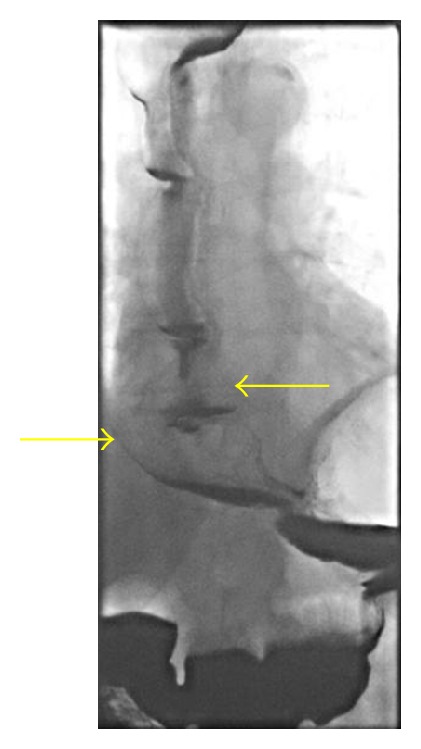
Barium swallow showing dilated lower oesophagus (no achalasia cardia).

Flexible oesophogastroduodenoscopy was performed the following day to examine the lower oesophagus. This revealed a grossly dilated and tortuous oesophagus (Figure 2) without the presence of an oesophageal stricture. Barium swallow was also performed and demonstrated a uniformly dilated, atonic oesophagus without evidence of achalasia (Figures 3 and 4).

The placement of the tracheostomy resulted in the patient being discharged several days after safe removal and adequate closure of the surgical site.

## 3. Discussion

Stridor secondary to tracheal compression caused by megaesophagus is rare [1].

Megaesophagus is believed to arise as a result of failure of normal synchronised peristalsis causing oesophageal dilatation [2].

The most common oesophageal motility disorder leading to megaesophagus is achalasia, a neurological disease characterised by degeneration of ganglion cells of the inhibitory intramural myenteric plexus. This leads to an inability of muscle contraction in the distal two-thirds and concomitant failure of relaxation of the lower oesophageal sphincter which can cause retention of food bolus [3, 4]. These patients present with a well-recognised and long standing history of dysphagia, particularly to liquids. This associated feature may therefore alert the discerning clinician to a correct diagnosis. In cases of achalasia as the primary cause of an obstructing megaesophagus, these patients usually present subacutely with slowly increasing airway compromise. Decompression of the oesophagus via passage of a wide-bore nasogastric tube has been reported to be a simple and highly successful method of treatment, with the patients improving within a matter of minutes [1].

Other previously recognised causes of oesophageal motility disorders that have been documented, particularly amongst the elderly population, are those secondary to diabetic autonomic neuropathy and Parkinson's disease [5, 6].

We present a unique case of an obstructing megaesophagus secondary to an acquired idiopathic dysmotility disorder, which had not been reported previously. Atypical features of this case differentiate this unusual condition from that of the described case reports secondary to achalasia, including the absence of preceding dysphagia, very acute compromise of the airway, and the notable swelling in the neck with venous congestion of the neck veins. Simple decompression with an orogastric tube would be impossible due to impacted solid food bolus, and intensive manual disimpaction was required.

Oesophageal motor dysfunction is a common finding in the elderly, but its aetiology remains largely unknown. One study examined the histology of Auerbach's plexus and oesophageal smooth muscle in autopsy material from 24 young and old deceased people. It concluded that there was a significant decrease in the density of ganglion cells per square centimetre in the elderly compared with that of the young population. The study also discovered that there was no difference between the degree of thickness of the oesophageal smooth muscle. This rejected previous views that oesophageal dysmotility in the elderly might be due to small muscle atrophy [7].

## 4. Conclusion

Airway obstruction secondary to megaesophagus is a rare complication, which requires urgent management. In cases of an obstructing megaesophagus secondary to achalasia, recognition from the history and chest radiograph findings may allow simple and effective treatment with wide-bore nasogastric (NG) or orogastric (OG) tube placement.

However, in the instance of obstructing megaesophagus secondary to idiopathic oesophageal dysmotility, a secure airway must be established, as decompression with an NG/OG tube will not suffice to relieve the obstruction. If an anaesthetist confident in awake fibre-optic intubation is available and can secure the airway then emergency tracheostomy under local anaesthesia is not necessary prior to oesophageal food bolus disimpaction.

With hindsight, given that manual disimpaction of the oesophagus resulted in immediate resolution of the airway compromise in this case, it may have been possible to avoid a temporary tracheostomy and the delayed discharge as a result of its placement. However, in every case, if there is any concern with regards to securing a patient's airway safely, a tracheostomy cannot be dismissed.

## 5. Summary

- Acquired idiopathic dysmotility disorder is a common finding in the elderly. One study concluded that there is a significant decrease in the density of ganglion cells per square centimetre in the oesophageal smooth muscle in the elderly population compared with the young population.
- However, it rarely causes obstructing megaesophagus. In this condition, stridor occurs when an impacted food bolus causes displacement of the upper oesophagus behind the pharynx compressing the airway. Secondary venous congestion and oedema further compromise the airway.
- We feel that obstructing megaesophagus should be managed with awake fibreoptic intubation, where possible, with subsequent disimpaction of oesophageal food bolus which will then relieve the airway obstruction.
- If the airway cannot be secured, then emergency tracheostomy under local anaesthetic must be performed prior to disimpaction of food bolus.
